# Cell Cycle Progression Influences Biofilm Formation in Saccharomyces cerevisiae 1308

**DOI:** 10.1128/spectrum.02765-21

**Published:** 2022-06-07

**Authors:** Ying Jiang, Caice Liang, Wei Zhao, Tianpeng Chen, Bin Yu, Anqi Hou, Jiaqing Zhu, Tao Zhang, Qingguo Liu, Hanjie Ying, Dong Liu, Wenjun Sun, Yong Chen

**Affiliations:** a National Engineering Research Center for Biotechnology, College of Biotechnology and Pharmaceutical Engineering, Nanjing Tech University, Nanjing, China; b State Key Laboratory of Materials-Oriented Chemical Engineering, College of Biotechnology and Pharmaceutical Engineering, Nanjing Tech University, Nanjing, China; c School of Chemical Engineering and Energy, Zhengzhou University, Zhengzhou, China; d Nanjing Hi-Tech Biological Technology Research Institute Co., Ltd, Nanjing, China; University of Debrecen

**Keywords:** cell cycle, biofilm, *S. cerevisiae*, *CLN3*, *SIC1*, *ACE2*

## Abstract

Biofilm-immobilized continuous fermentation is a novel fermentation strategy that has been utilized in ethanol fermentation. Continuous fermentation contributes to the self-proliferation of Saccharomyces cerevisiae biofilms. Previously, we successfully described the cell cycle differences between biofilm-immobilized fermentation and calcium alginate-immobilized fermentation. In the present study, we investigated the relationship between biofilm formation and the cell cycle. We knocked down *CLN3*, *SIC1*, and *ACE2* and found that Δ*cln3* and Δ*sic1* exhibited a predominance of G_2_/M phase cells, increased biofilm formation, and significantly increased extracellular polysaccharide formation and expression of genes in the *FLO* gene family during immobilisation fermentation. Δ*ace2* exhibited a contrasting performance. These findings suggest that the increase in the proportion of cells in the G_2_/M phase of the cell cycle facilitates biofilm formation and that the cell cycle influences biofilm formation by regulating cell adhesion and polysaccharide formation. This opens new avenues for basic research and may also help to provide new ideas for biofilm prevention and optimization.

**IMPORTANCE** Immobilised fermentation can be achieved using biofilm resistance, resulting in improved fermentation efficiency and yield. The link between the cell cycle and biofilms deserves further study since reports are lacking in this area. This study showed that the ability of Saccharomyces cerevisiae to produce biofilm differed when cell cycle progression was altered. Further studies suggested that cell cycle regulatory genes influenced biofilm formation by regulating cell adhesion and polysaccharide formation. Findings related to cell cycle regulation of biofilm formation set the stage for biofilm in Saccharomyces cerevisiae and provide a theoretical basis for the development of a new method to improve biofilm-based industrial fermentation.

## INTRODUCTION

In the 1970s, bacteria were described to be single free-floating microorganisms by Robert Koch (the father of modern microbiology) in his seminal research (1). At that time, scientists had studied many deadly bacteria and developed bactericides to kill them. However, the emergence of drug-resistant bacteria, and the difficulty in killing them, have made studying bacterial lifestyles increasingly important. It is known that bacteria have the ability to form dense and complex microbial aggregates that can adhere to biological and abiotic surfaces (1). These aggregates contribute to defending against bactericides and were named “biofilm” by Coston in 1978.

Among fungi, *S.cerevisiae* has been introduced as an attractive model for biofilm studies because it is genetically tractable and has several properties such as short growth cycles and ease of culture (2, 3). The results suggested that *S.cerevisiae* can initiate biofilm formation and that the formation of *S.cerevisiae* biofilm might consist of two parts: adhesion to the medium and further maturation (3). Biofilms are microbial communities composed of cells in an extracellular matrix and are attached to a surface. The composition of the biofilm matrix varies with different microorganisms and under different growth conditions. However, the biofilm matrix is generally composed of extracellular polysaccharides, proteins, and nucleic acids (4), which support the mechanical stability of biofilms, mediate adhesion to surfaces, and form a cohesive three-dimensional polymer network (5).

Fungi have a surprising ability to adhere and grow on distinct substrates or hosts. For example, S. cerevisiae forms biofilms, which is a major concern in food safety. S. cerevisiae forms biofilms in wine stocks, which may lead to defects such as turbidity or blurring, sediment production, and off-flavors (6). Additionally, biofilms have high environmental tolerances and are difficult to eradicate with the treatment of current antifungal drugs. Therefore, current research has focused on studying new approaches to prevent biofilm formation. For example, inhibitory and dispersive antibacterial agents, including peppermint essential oils (7), thyme essential oils (8), and small molecules, such as aldehydic terpenes (9) and tannins (10), have been used in an attempt to inhibit biofilm formation. Contrastingly, biofilms show great advantages in industrial applications because of their strong environmental resistance and stability. Li et al. (11) used biofilms to conduct continuous ethanol fermentation and discovered that the biofilm fermentation period was short and stabilized after 4 h, which was approximately one-quarter that of free fermentation. The researchers compared the fermentation capacity of immobilized and free fermentation models, utilizing cassava hydrolysate, and found that the starch utilization of immobilized cells was 2.1% higher than that of free cells under the same fermentation conditions (12). Moreover, Liang et al. (13) found that yeasts inside biofilms and calcium alginate beads have distinct preferences during continuous fermentation, with the cell cycle of biofilm-immobilized cells being continuously enriched in the G_2_/M phase as batch times grew, while calcium alginate-immobilized cells were predominantly in the G_1_/G_0_ phase.

**FIG 1:**
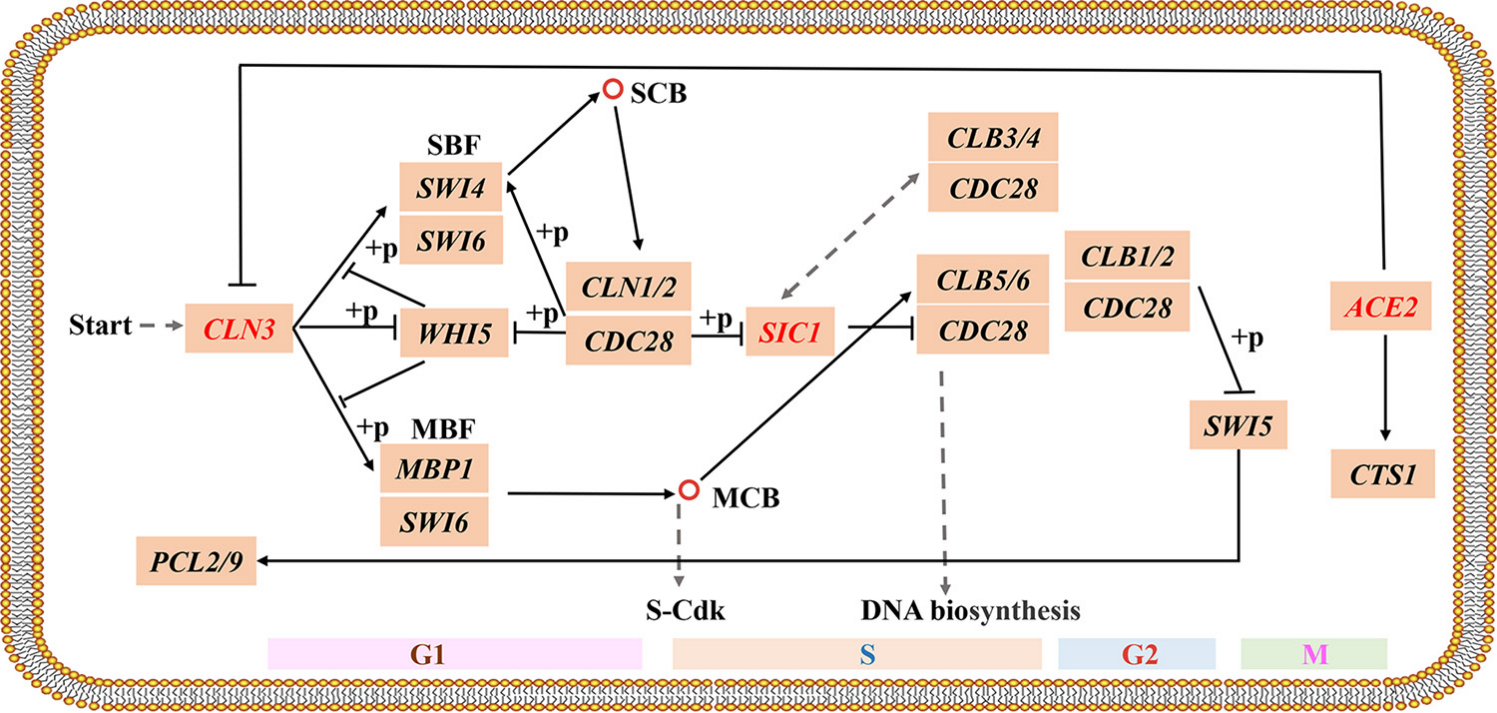
Schematic diagram of the cell cycle pathway in Saccharomyces cerevisiae.

The cell cycle is a complex process that involves numerous regulatory proteins that direct the cell through a specific sequence of events, culminating in mitosis and two daughter cells (14). The cell cycle can be divided into four phases: G_1_/G_0_, S, G_2_, and M phases. The G_1_ phase is a period of growth, and cells in the G_1_ phase synthesize cellular macromolecules, including proteins, RNA, and cell membranes (15). When the cell cycle begins, Cln3-Cdk1 phosphorylates *WHI5* and *STB1* and activates the G_1_-phase cyclins Cln1p and Cln2p, which allows cells to transition from the G_1_ phase to the S phase (16). The S phase is a period of DNA synthesis (17). *SIC1* is heavily degraded at the onset of the S phase, which leads to the initiation of DNA synthesis through a switch mechanism of Cdk1/Cln1-mediated multisite phosphorylation, thereby derepressing S-phase CDKs (Cdk1/Clb5, 6) and allowing the cell to enter the S phase (18). The G_2_ phase is the interval between the completion of DNA synthesis and mitosis, and the M phase is the mitotic phase, marked by the production of bipolar mitotic spindles, sister chromatid separation, and cytokinesis (17). Ace2p regulates the transcription of *CTS1* (a chitinase) gene and mediates its regulatory function in postmitotic mother-daughter cell separation (19) (Fig. 1).

Liang et al. (13) revealed that there is a specific connection between the cell cycle and biofilm formation, but it remains unknown how the cell cycle interacts with the biofilm and how they influence each other. The present study focused on this aspect and aimed to understand the relationship between biofilm formation and the cell cycle. We knocked down *CLN3*, *SIC1*, and *ACE2* to evaluate the resulting changes in the cell cycle and biofilm formation.

## RESULTS

### The ability to form biofilm is influenced by cell cycle changes.

**FIG 2:**
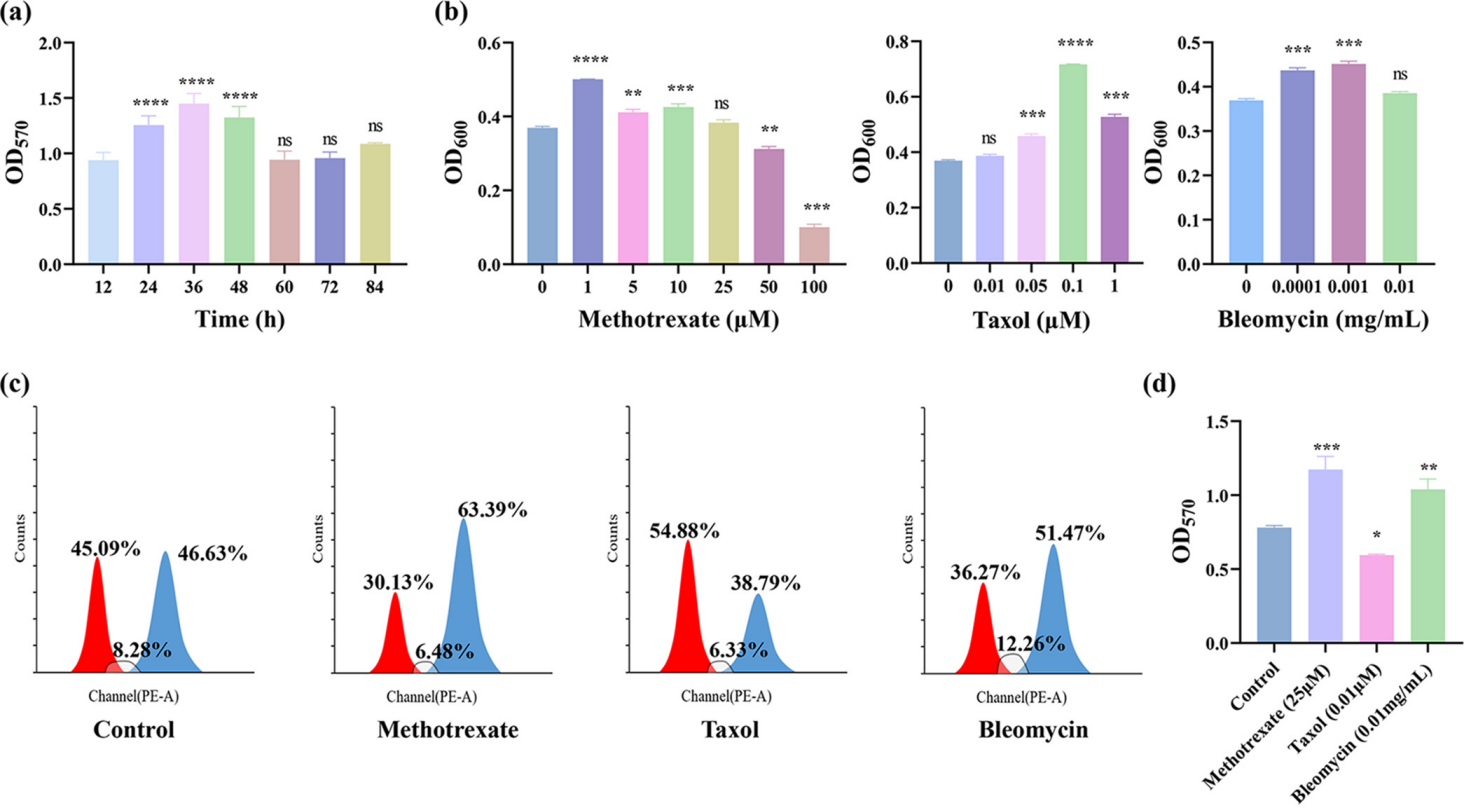
Changes in cell cycle after drug culture, the suitable time of biofilm growth, drug concentrations that have no effect on cell growth, changes in biofilm after drug incubation. (a) Changes in cell cycle after culturing wild type (WT) with 25 μM methotrexate, 0.01 μM taxol, 0.01 mg/mL bleomycin for 36 h at 200 rpm, 30°C. Red, proportion of cells in the G_1_/G_0_ phase; blue, proportion of cells in the G_2_/M phase; gray-shaded, proportion of cells in the S phase. (b) Biofilm formation ability of WT at 12, 24, 36, 48, 60, 72, and 84 h. (c) Methotrexate was selected in a concentration gradient of 100, 50, 25, 10, 5, and 1 μM; taxol was selected in a concentration gradient of 5, 1, 0.05, and 0.01 μM; and bleomycin was selected in a concentration gradient of 0.01, 0.001, 0.0001 mg/mL, and the cells were cultured for 36 h at 200 rpm, 30°C under these conditions. (d) Changes of biofilm after incubating WT with 25 μM methotrexate, 0.01 μM taxol, 0.01 mg/mL bleomycin for 36 h at 200 rpm, 30°C. The error bars represent SD. ****, *P* > 0.05; ***, *P* < 0.001; **, *P* < 0.01; *, *P* < 0.05; ns, not significant.

To investigate the relationship between biofilm formation and the cell cycle, we used some drugs to induce the cells to remain in a specific phase of the cell cycle. Methotrexate, an inhibitor of folate reductase, inhibits dihydrofolate reductase and prevents the reduction of dihydrofolate to the physiologically active tetrahydrofolate, thus inhibiting purine nucleotide and pyrimidine biosynthesis, resulting in the inhibition of DNA biosynthesis (20), which plays a major role in the S phase of the cell cycle. Additionally, taxol is able to induce elevated levels of reactive oxygen species (ROS) in S. cerevisiae, thereby delaying cell cycle progression and possibly leading to cell accumulation in the G_1_/G_0_ phase (21, 22). Moreover, bleomycin produces single- and double-strand scissions in DNA leading to G_2_/M arrest in checkpoint-proficient cells (23). The proportion of cells in the G_2_/M phase increased by 36% in the methotrexate-treated group, that of cells in the G_1_/G_0_ phase increased by 22% in the taxol-treated group, and that of bleomycin-treated cells showed a 10% increase in the G_2_/M phase (Figure 2a). The results suggested that there might be a correlation between biofilm formation and the cell cycle because biofilm formation is affected by cell cycle changes (methotrexate and bleomycin enhanced biofilm formation, whereas taxol reduced biofilm formation). We determined that the suitable time of biofilm growth was 36 h (Figure 2b), and the valid drug concentrations that worked were 25 μM methotrexate, 0.01 μM taxol, and 0.01 mg/mL bleomycin (Figure 2c). Surprisingly, both methotrexate and bleomycin promoted biofilm formation in cells cultured for 36 h, whereas taxol reduced biofilm formation (Figure 2d).

### The effects of *SIC1*, *CLN3,* and *ACE2* on the cell cycle.

**FIG 3:**
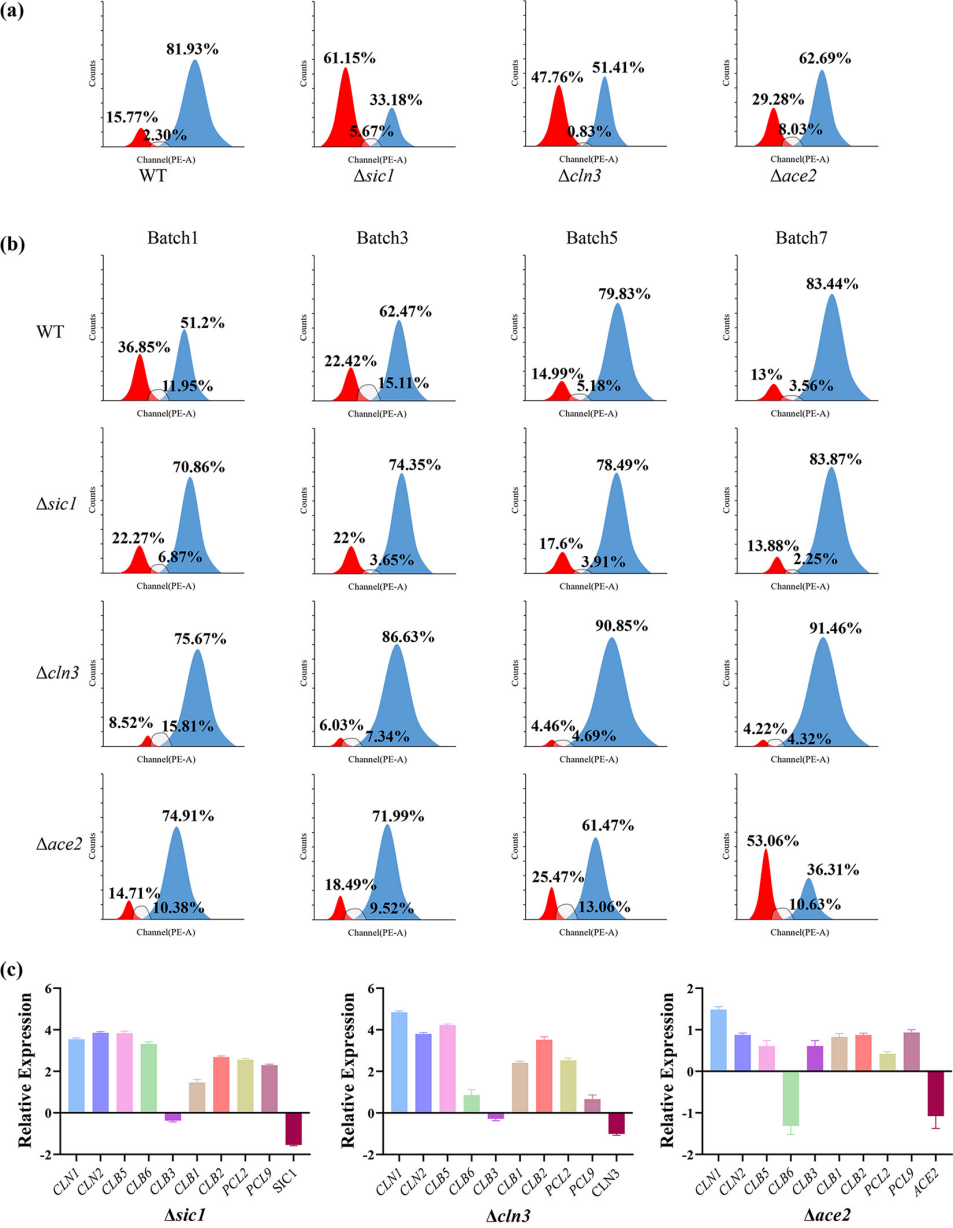
Effects of deletion of genes *SIC1*, *CLN3*, and *ACE2* on cell cycle. (a) The distribution of cell cycle of WT/Δ*sic1*/Δ*cln3*/Δ*ace2* in seed fluid. Red, proportion of cells in the G_1_/G_0_ phase; blue, proportion of cells in the G_2_/M phase; gray-shaded, proportion of cells in the S phase. (b) Cell cycle changes on vectors in immobilized fermentation during batch growth. Red, proportion of cells in the G_1_/G_0_ phase; blue, proportion of cells in the G_2_/M phase; gray-shaded, proportion of cells in the S phase. (c) Differences in the expression of cycle-related genes in Δ*sic1*/Δ*cln3*/Δ*ace2*, all statistical analyses were compared with WT. The error bars represent SD.

To verify the role of *SIC1*, *CLN3*, and *ACE2* in the cell cycle under the culture conditions of this experiment (200 rpm, 30°C), Δ*sic1*, Δ*cln3*, Δ*ace2*, +*pSIC1*, +*pCLN3*, and +*pACE2* strains were constructed. Flow cytometry was used to measure the distribution of the cell cycle phases of several strain-modified cell cycle-related genes in seed fluid (Figure 3a), immobilized cells (Figure 3b), and free cells in the fermentation broth (Fig. S1). Quantitative real-time PCR (qRT-PCR) assay was used to analyze the expression of related genes (Figure 3c). In the seed fluid, the cell cycle ratio of several modified strains differed from that of the wild type (WT). Independent of free cells and immobilized cells, Δ*sic1* and Δ*cln3* had large proportions of cells in the G_2_/M phase, which gradually increased as the fermentation batches grew, whereas Δ*ace2* led to an increased number of cells in the G_1_/G_0_ phase. This result supported the findings of the flow cytometry experiment. In parallel, we measured the apoptosis rate of the sample cells. We found that the apoptosis rate of Δ*sic1* was higher than that of WT, and the apoptosis rates of Δ*ace2* and Δ*cln3* were both lower than that of WT, implying that Δ*cln3* and Δ*ace2* grew slightly better than WT, and Δ*sic1* grew slightly weaker (Fig. S2). Δ*sic1* and Δ*cln3* preferentially expressed *CLB6*, with an increased expression of approximately 4-fold in Δ*sic1*. However, the knockout strain displayed a decrease in *CLB6* expression in Δ*ace2*. Furthermore, *CLB3* expression was downregulated in Δ*sic1* and Δ*cln3* but upregulated by 1-fold in Δ*ace2*.

### The effects of *SIC1*, *CLN3*, and *ACE2* on biofilms.

**FIG 4:**
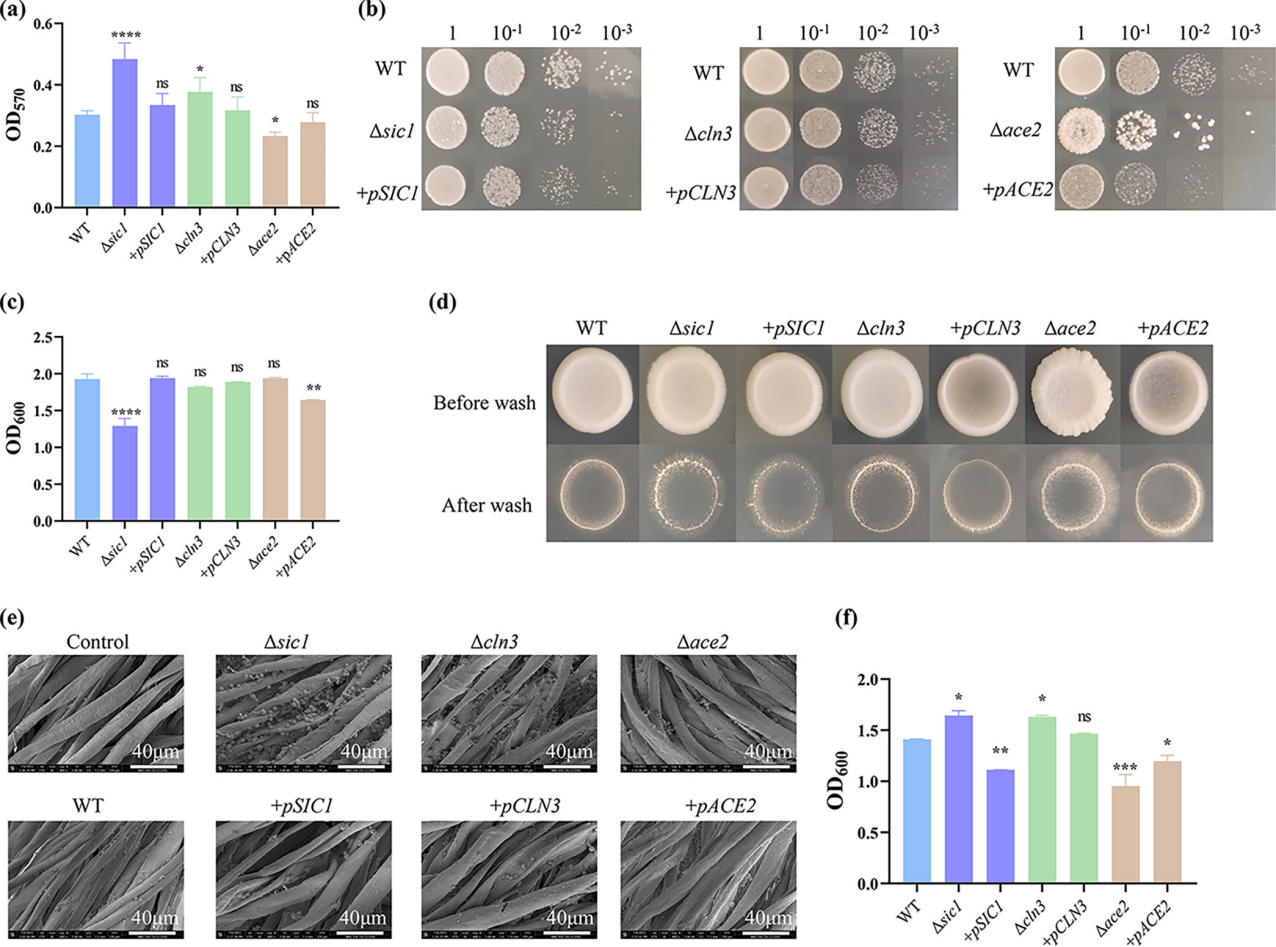
Effects of deletion of genes *SIC1*, *CLN3*, and *ACE2* on biofilms. (a) Biofilm formation ability of WT, Δ*sic1*, +*pSIC1*, Δ*cln3*, +*pCLN3*, Δ*ace2*, and +*pACE2*. (b) Growth ability of WT and six mutants. (c) Growth ability was expressed as optical density at 600 nm (OD_600_). (d) Standard plate-wash assay of WT, Δ*sic1*, +*pSIC1*, Δ*cln3*, +*pCLN3*, Δ*ace2*, and +*pACE2*. (e) Scanning electron microscopy (SEM) images of biofilm formed on cotton fibers with control, WT, Δ*sic1*, Δ*cln3*, Δ*ace2*, +*pSIC1*, +*pCLN3*, and +*pACE2* after 12 h of fermentation. Scale bar, 40 μm. (f) After fermentation, the OD_600_ of biofilm cells on the carrier was detected. The error bars represent SD. ****, *P* > 0.05; ***, *P* < 0.001; **, *P* < 0.01; *, *P* < 0.05; ns, not significant.

Compared with that in the WT, the biofilm formation of Δ*sic1 and* Δ*cln3* increased by 51% and 16.5%, respectively, while that of Δ*ace2* decreased by 10.9%. As expected, there was little difference in biofilm formation in the back-supplemented strains (Figure 4a) and the WT.

The growth of the strain on the plate (Figure 4b) and the optical density (OD) values (Figure 4c) demonstrated that deletion of *CLN3* and *ACE2* had no significant effect on the growth of the strains, whereas deletion of *SIC1* resulted in slightly weaker growth of the mutant strain on the YPD plates than the WT.

Subsequently, a standard plate-wash assay was performed to assess the invasive growth ability of the strains. The number of colonies formed by Δ*ace2*, Δ*cln3*, and Δ*sic1* remained remarkably higher than that formed by the WT (Figure 4d).

Images of these strains on cotton fibers were acquired using scanning electron microscopy (SEM) (Figure 4e) and observed that Δ*sic1 and* Δ*cln3* notably persisted on cotton fibers, while the other strains did not differ from the WT. This result was consistent with the semi-quantitative analysis of the biofilms of each strain on cotton fibers (Figure 4f).

### *SIC1*, *CLN3*, and *ACE2* regulate biofilm formation by influencing cell adhesion and extracellular polysaccharide production.

**FIG 5:**
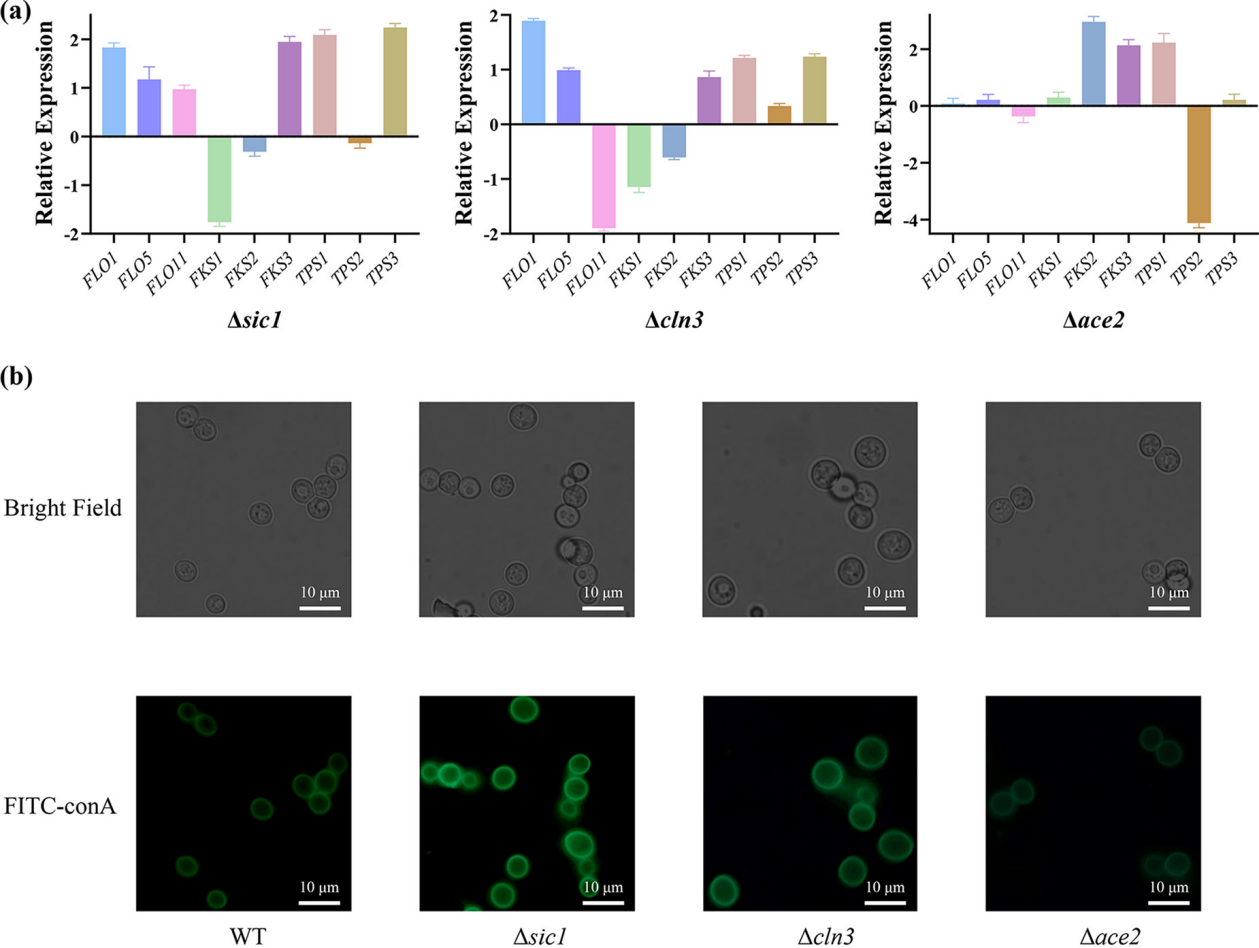
Effects of *SIC1*, *CLN3*, and *ACE2* genes deletions on extracellular polysaccharide production. (a) Quantitative real-time-PCR analysis of relative expressions of *FLO1*/*5*/*9* and *FKS1*/*2*/*3* and *TPS1*/*2*/*3* genes in Δ*sic1*, Δ*cln3*, and Δ*ace2*, all statistical analyses were compared with WT. The error bars represent SD. (b) FITC-conA fluorescent staining result of WT, *Δsic1*, Δ*cln3*, and Δ*ace2*. Scale bar, 10 μm.

As glucan and trehalose are indispensable components of biofilms, qRT-PCR was conducted to determine the expression levels of glucan synthase (*FKS1*/*2*/*3*) in the cell wall and trehalose synthase (*TPS1*/*2*/*3*) in the cytoplasm as well as genes in the *FLO* gene family (Figure 5a), to further explore the mechanisms underlying biofilm formation. The deletion of both *SIC1 and CLN3* resulted in a 2-fold and 1-fold increase in the expression of *FLO1* and *FLO5*, respectively, whereas the expression of *FLO11* decreased by almost twice in Δ*cln3* but increased approximately 2-fold in Δ*sic1*. Deletion of both *SIC1* and *CLN3* resulted in reduced expression of *FKS1* and *FKS2* and increased expression of *FKS3*. Deletion of *SIC1* and *CLN3* also led to the upregulation of *TPS1* and *TPS3*. In contrast, Δ*ace2* expressed one-quarter as much *TPS2* and slightly less *FLO11*, while *FKS2*, *FKS3*, and *TPS1* translated slightly more corresponding proteins.

To verify the results of this experiment, we dyed the extracellular polysaccharides with FITC-ConA (Figure 5b). The results indicated that Δ*sic1* and Δ*cln3* displayed more intense fluorescence than the WT, while the fluorescence of Δ*ace2* was notably less intense. These findings show that extracellular polysaccharide formation is increased following the knockdown of *SIC1* and *CLN3* and decreased following *ACE2* knockdown.

## DISCUSSION

Biofilm-immobilized fermentation, a new immobilized strategy, often results in increased fermentation abilities because of its higher tolerance and fermentation rate. In the process of biofilm formation, the internal cells continuously progress to the G_2_/M stage, which indicates that the biofilm has its own mechanisms in which it can modulate cell activity and biofilm structure. To determine the relationship between biofilm formation and the cell cycle, we evaluated the ability of cells to form a biofilm by controlling the progression of the cell cycle using 25 μL methotrexate, 0.01 μL taxol, and 0.01 mg/mL bleomycin in the initial step. Crystal violet (CV) staining assay showed that methotrexate and bleomycin promoted biofilm formation, but taxol did not. An increase in the proportion of cells in stage G_2_/M of the cell cycle was conducive to the formation of biofilm, while an increase in the proportion of cells in the G_1_/G_0_ stage had an adverse effect on the biofilm formation system.

Methotrexate-treated cells showed a 36% increase in the proportion of cells in the G_2_/M phase. Taxol-treated cells exhibited a 22% increase in cells retained in the G_1_/G_0_ phase, and those treated with bleomycin showed a 10% increase in cells in the G_2_/M phase. The results illustrated that there might be a relationship between biofilm formation and the cell cycle because biofilm formation was affected by cell cycle changes (methotrexate and bleomycin enhanced biofilm formation, whereas taxol reduced it).

In the cell cycle, *CLN3* is the most upstream regulatory gene involved in the G_1_/S transition (START) and directly catalyzes the cell cycle (24). *SIC1* is heavily degraded at the beginning of the S phase, and the inhibition of CDKs (Clb5,6-Cdk1) is relieved through the switching mechanism of multisite phosphorylation, mediated by Cln1-Cdk1. This leads to the initiation of DNA synthesis and allows cells to enter the S phase (25). Clb3p is a cyclin that is expressed in the S/G_2_ phase (26). Clb1p and Clb2p are expressed in the G_2_ phase (18), whereas Pcl2p and Pcl9p are expressed in the M/G_1_ phase (27, 28). Ace2p is a transcription factor required for septum decomposition after cytokinesis (19).

To build on this, we constructed deletion and complement mutants of *SIC1*, *CLN3*, and *ACE2* and tracked the cell cycle changes in yeast cells in biofilms during ethanol fermentation. We found that as the fermentation batches grew, the proportion of cells in the G_2_/M phase in Δ*sic1 and* Δ*cln3* was remarkably large and gradually increased both on vectors and in the fermentation broth, while the proportion of cells in the G_1_/G_0_ phase in Δ*ace2* gradually increased. Surprisingly, the fraction of cells in G_2_/M phase of cell cycle of Δ*sic1* strain and WT was the same postbatch 3. We suspected that the growth capacity of Δ*sic1* was weaker than that of WT (according to the data in Figure 4b). With the progress of fermentation, the aging of cells and the decline of metabolic capacity led to its inconspicuous advantages compared with the WT.

The results of the qRT-PCR analysis showed that the expression of *CLB5*/*CLB6* in Δ*sic1* was approximately 4-fold greater, which is consistent with the literature (18). The expression of *CLB3* was slightly reduced in Δ*sic1* and Δ*cln3*, suggesting that knockout of *SIC1*, which regulated S-phase, and *CLN3*, which regulated G_1_-phase, had an effect on G_2_-phase cell activity. While in Δ*sic1* and Δ*cln3*, the expression of *CLN1* and *CLN2* was still high. Although *CLN3* plays a crucial role in the cell cycle, cells without *CLN3* are still able to enter the cell cycle, as there are still other mechanisms that promote cell cycle progression, such as *Bck2*, leading to high expression of *CLN1/2* (29). In addition, in Δ*sic1*, G_1_-phase cells were still active, so there was a high expression of *CLN1/2*. In Δ*ace2*, the expression levels of all genes were low, and *CLB6* was downregulated. We hypothesized that this result was related to cytokinesis, which was regulated by *ACE2*. When *ACE2* was knocked out, the cells could not enter the next cell cycle smoothly, and hence, the expression of genes related to the cell cycle was diminished.

The CV staining assay illustrated that Δ*sic1 and* Δ*cln3* greatly enhanced the ability of S. cerevisiae 1308 cells to form biofilms; however, Δ*ace2* weakened this ability. *FLO1* and *FLO5* confer cell-cell viscosity and contribute to flocculation (30). *FLO11* encodes a flocculating protein that enhances cell-matrix adhesion and could play a role in development of biofilm in liquid medium (31, 32). The results of the qRT-PCR analysis revealed that the expression levels of *FLO1* and *FLO5* were upregulated in Δ*sic1 and* Δ*cln3*, while the expression of *FLO11* was upregulated by 1-fold in Δ*sic1* but downregulated by 2-fold in Δ*cln3.* These results indicate that Δ*cln3* and Δ*sic1* promote cell-cell adhesion, with Δ*sic1* also promoting cell-medium adhesion, all of which increase the capacity for biofilm formation.

Plate-wash assay was to test the ability of invasive growth of different strains. The ability of invasive growth depends on cell-substrate adhesion, which plays a vital role in biofilm formation (31). In this study, Δ*sic1 and* Δ*cln3* formed more colonies on YPD plates than did WT. This result was consistent with their promotion of biofilm formation. However, Δ*ace2* also formed prominent colonies. A review of the literature revealed that in the generally nonpathogenic yeast S. cerevisiae, deletion of *ACE2* resulted in increased pseudohyphal growth and invasion of agar, which was consistent with our experimental results (33, 34). Extracellular polysaccharides are the main components of most microbial biofilms (35). During biofilm formation, the secretion of extracellular polysaccharides may play a role in promoting the formation of complex biofilm structures (36). The results of the qRT-PCR showed downregulation of *FKS1* expression in Δ*sic1 and* Δ*cln3*, which led to a decrease in β-glucan but an increase in chitin and mannoprotein levels in the cell wall (37). In addition, fluorescence staining of extracellular polysaccharides showed that the polysaccharide content of Δ*sic1 and* Δ*cln3* increased significantly, while that of Δ*ace2* decreased. Therefore, extracellular polysaccharide content also influences the formation of cellular biofilms.

### Conclusion.

In conclusion, this study focused on the effects of the cell cycle on biofilm formation in S. cerevisiae. The ability of S. cerevisiae to form biofilm was stronger when cells accumulated in the G_2_/M stage but weaker when cells were in the G_1_/G_0_ phase. Δ*sic1* and Δ*cln3* increased the proportion of cells in G_2_/M phase of the cell cycle, which in turn increased extracellular polysaccharide formation and the expression of *FLO* genes. Together, this affected the ability of the cells to form biofilms. In industrial fermentation, biofilm formation can be increased by preventing the cell cycle from entering a stationary state (G_0_), which improves the antistress ability of cells and enhances cell cycle progression under stressful conditions for sustained fermentation (38). The important factors identified in this study may be used to regulate biofilm formation during immobilized fermentation. On the other hand, the temporal changes of the cell cycle in biofilms imply that biofilms may have their own structures and perform different functions due to cell cycle progression with time, which we will do in the future. In addition, to strongly demonstrate that the cell cycle and biofilm are linked, it is necessary to carry out additional experiments, such as extracellular polysaccharide levels, expression of enzymes involved in polysaccharide synthesis, and cell adhesion proteins, for drug-treated cells as shown for genetic mutants in the future.

## MATERIALS AND METHODS

### Yeast strains and growth conditions.

This study was carried out with S. cerevisiae 1308 (39), which was cultured on YPD plates (1% yeast exact, 2% peptone, 2% glucose, and 2% agar). The fermentation medium was formulated with glucose (55 g/L), peptone (4 g/L), (NH_4_)_2_SO_4_ (4 g/L), yeast extract (3 g/L), KH_2_PO_4_ (3 g/L), MgSO_4_ (0.5 g/L), ZnSO_4_-7H_2_O (0.05 g/L), and FeSO_4_-7H_2_O (0.05 g/L) (39). The mutant strains were selected by adding to YPD plates with the antibiotic G418 sulfate at a final concentration of 500 μg/mL.

The seed fluid was cultured in 250-mL flasks containing 100 mL YPD liquid medium at 30°C and 200 rpm (ZQTY-70N, Shanghai Zhichu). Immobilized fermentation was performed in 250-mL flasks with 100 mL fermentation medium and approximately 4 g dry cotton fiber at 200 rpm, 35°C. Repeated batch fermentation was performed with the immobilized culture, by removing the supernatant and adding fresh medium following the depletion of residual glucose (<5 g/L). Samples were drawn from every flask at the end of each fermentation batch (39).

### Drug experiments.

When the cells were grown to OD_600_ of 1, drugs were added. Methotrexate was selected for a concentration gradient of 100, 50, 25, 10, 5, and 1 μM; taxol for a concentration gradient of 5, 1, 0.05, and 0.01 μM; and bleomycin for a concentration gradient of 0.01, 0.001, and 0.0001 mg/mL, and the cells were incubated for 36 h at 200 rpm, 30°C under these conditions.

### Construction of mutant and complemented strains.

S. cerevisiae strains mutants were constructed by deleting corresponding genes in S. cerevisiae 1308 using CRISPR/Cas9 (40). Competent S. cerevisiae 1308 cells produced using the sorbitol method were transformed with modified plasmid and linear repair DNA via electroporation (Bio-Rad, Hercules, CA, USA) at 1.5 kV, 25 mF with a 200-Ohm pulse controller.

*CLN3*, *SIC1*, and *ACE2* were amplified from the genome of S. cerevisiae 1308 by corresponding primers, purified, and inserted into plasmid pYX212 using ClonExpress one-step cloning kit (Vazyme Biotech, Nanjing, China). G418 sulfate was used to select the plasmid carrying the above genes into the knockout mutant strain. The PCR primers used are shown in Table S1.

### RNA preparation, cDNA library construction, and qRT-PCR analysis.

After 12 h of immobilized fermentation, the fermentation liquid was discarded, and the cotton fiber was cleaned three times with 100 mL phosphate-buffered saline (PBS, pH 7.4) buffer solution (1.44 g/L Na_2_HPO_4_, 0.2 g/L KCl, 8 g/L NaCl, and 0.24 g/L KH_2_PO_4_). Cells were collected and immediately frozen in liquid nitrogen (13). Three biological replicates were used for each condition (41). Total RNA was extracted using a column-based total RNA extraction kit (TaKaRa, China).

cDNA libraries for qRT-PCR were constructed using HIScript II Q RT SuperMix for qPCR (+gDNA wiper, Vazyme, Nanjing, China). The primers were designed using sequences available in the GenBank database of the National Center for Biotechnology Information (NCBI) as references. The genes and primers used for analysis are listed in Table S2.

Quantitative real-time PCR assays were performed using the StepOnePlus real-time PCR system (Applied Biosystems) and 2× ChamQ Universal SYBR qPCR Master Mix (Vazyme, China). Reactions and calculations were performed according to the manufacturer’s instructions, with three technical replicates for each sample and one negative control without cDNA. We used a false discovery rate threshold of ≤ 0.001 and an absolute value of the Log_2_ ratio ≥ 1 as criteria for assessing the significance of differential gene expression (13).

### Biofilm forming capacity on plastics.

In order to evaluate the ability of yeast strains to form biofilms, the CV staining assay was performed as previously described with minor modification (31). S. cerevisiae strains were cultured for 12 h in YPD medium, after which cells were collected and washed with PBS. A volume of 20 μL of this culture at OD_600_ = 1 was transferred to a 96-well plate containing 180 μL of fermentation medium per well. Plates were incubated at 30°C for 36 h, and then, free fermentation cells were washed with PBS and 1% crystal violet. Excess dye was then removed by washing with distilled water. Glacial acetic acid (200 μL) was added, and the mixture was agitated at 150 rpm for 30 min at room temperature. Absorbance at 570 nm was measured using a microplate reader (SpectraMax Paradigm, Molecular Devices, LLC, San Jose, CA, USA) (13).

### Standard plate-wash assay.

Each strain was grown on YPD plates (30°C) for 3 days. The growth of all strains was observed in this environment. Then, each plate was rinsed with running water until no colonies remained, and the prewash and after-wash condition of the strains on the plate was recorded.

### Growth capacity analysis.

Strains were cultured until cell density reached 1 and diluted to uniform cell density, followed by three 10-fold gradient dilutions with sterile water. Then, each diluent (2 μL) was dropped on the YPD plate and incubated at 30°C for 15 h to record the situation. At the same time, when the cell density reached 1, 10 μL of S. cerevisiae liquid was inoculated in 5 mL of liquid medium for 15 h at 200 rpm, 30°C and the absorbance at 600 nm was measured.

### SEM analysis.

Biofilm cells of strains were harvested after 12-h immobilized fermentation. Cotton fibers were washed twice with PBS buffer and stored at −80°C for 24 h. Biofilm cells were dried using a FreeZone 4.5 L Freeze Dry System (Labconco, Kansas City, MO, USA) and sputter-coated with gold. Images were acquired using SEM (SEM 4800, Hitachi, Japan) (11).

### Semiquantitative analysis of biofilm.

After the first fermentation (12 h), the supernatants were discarded and 100 mL of PBS was added to clean the unattached free cells on cotton fibers. Supernatant PBS was discarded and 100 mL of PBS was added again to sonicate (25°C, 40 Khz) the biofilm cells on cotton fibers for 3 h. Cell concentration was determined by spectral colorimetric measurements by measuring the optical density at 600 nm (OD_600_) (42).

### Flow cytometry.

When the cell density reached 3, the cells were inoculated into 250-mL flasks with cotton fibers for incubation. At the end of the fermentation, batch 1, 3, 5, and 7 cells were collected during immobilized fermentation and processed according to the instructions of the Cell cycle Kit (UElandy) for flow cytometric analysis (CytoFLEX, USA Beckman Coulter) (batch 1: 12 h; batch 2: 12 h; batch 3: 8 h; batch 4: 4 h; batch 5: 4 h; batch 6: 4 h; and batch 7: 4 h) as follows: after collecting the cells, centrifuge, carefully remove the supernatant, and add 1 mL of staining buffer to resuspend the cells; centrifuge, discard the supernatant, and add 1 mL of medium to resuspend the cell suspension; and add 4 μL of RedNucleusI staining buffer to each tube of cells, mix slowly and thoroughly, incubate for 20 min at room temperature, protected from light, and incubate with a flow cytometer at 638 nm for analysis of cellular DNA content using analytical software. In case of severe flocculation, multiple blown aspirations were performed to ensure cell dispersion, and the data were superimposed after multiple flow analyses to obtain the final results. Meanwhile, the apoptosis rate of the samples was tested with the apoptosis kit (UElandy).

### Fluorescence staining of extracellular polysaccharide.

First, yeast strains were cultured overnight using cells resuspended in YPD at OD_600_ of 1. Then, a cell coverslip was added to each well in 6-well plates, 3 mL YPD medium and 200 μL seed liquid were added, and the cultures were incubated at 30°C for 3 days. Free cells were washed off with PBS and the biofilm was fixed with 4% paraformaldehyde at 4°C for 30 min. The coverslips were washed twice with PBS, and then the extracellular polysaccharides in the biofilm were stained with 500 μL FITC-ConA (Sigma-Aldrich) (1 mg/mL) at room temperature for 30 min (43). The stained images were then taken with a fluorescence microscope (Mshot, MF52-N) under the same conditions (exposure time: 99 ms).

### Statistical analyses.

All experiments were performed at least in triplicate, and data represent the mean of three experiments. Differences between means were determined using the Student's *t* test and considering *P* < 0.05 as statistically significant.

### Data availability.

The authors promise the availability of supporting data.
