# Supplementary material for: Cell Cycle Progression Influences Biofilm Formation in Saccharomyces cerevisiae 1308
Source: Microbiol Spectr. 2022 Jun 7;10(3):e02765-21. doi: 10.1128/spectrum.02765-21 (PMC9241733; doi:10.1128/spectrum.02765-21)
Supplement: Supplemental file 1 — Supplemental material. Download spectrum.02765-21-s0001.pdf, PDF file, 0.2 MB [file spectrum.02765-21-s0001.pdf]

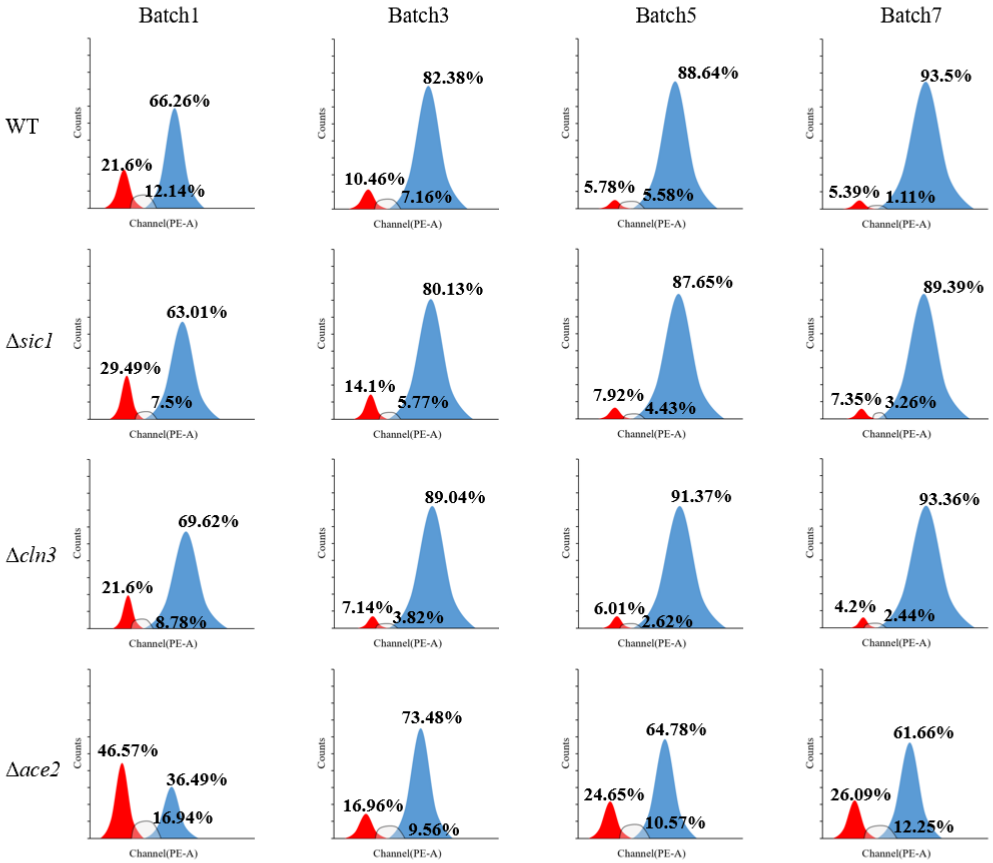

Fig. S1 Cell cycle changes in fermentation broth during batch growth. Red, proportion of cells in the G0/G1 phase; blue, proportion of cells in the G2/M phase; grey-shaded, proportion of cells in the S phase.

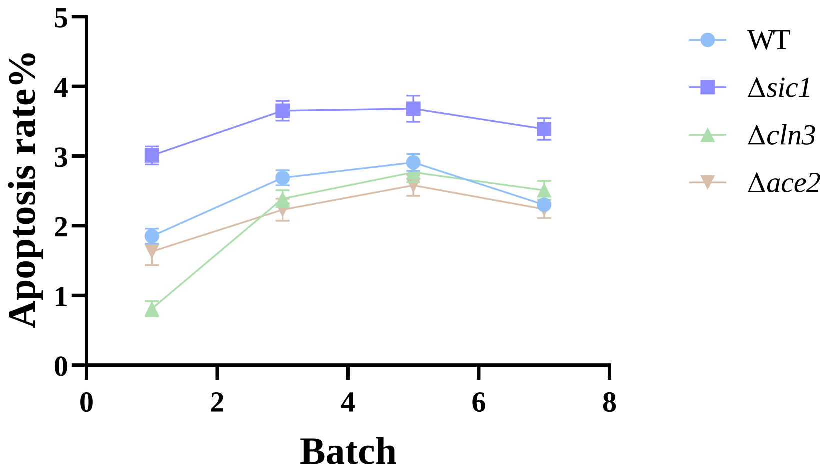

Fig. S2 Apoptosis rate of samples taken at the end of continuous immobilisation

fermentation.

Tables

Table S1 Primers used in this study and their sequences

Primer name	Primer sequence	Source
UP-CLN3-F	CGCCTGGCGTTGTCCTTTCTGCCCC	This work
UP-CLN3-R	AAATATCAGGACGAAGATGGTACATGGTTTCCAATTCTTG	This work
DOWN-CLN3-F	ACAAGAATTGGAAACCATGTACCATCTTCGTCCTGATATTT	This work
DOWN-CLN3-R	AATGAGGAAACAAGAGATAATTGGT	This work
UP-SIC1-F	TTCTGCATATTGCTTAACTG	This work
UP-SIC1-R	AACTTTTTTTTTCATTTCTTTTCGTGTAATAGTCCCTGC	This work
DOWN-SIC1-F	GCAGGGACTATTACACGAAAAGAAATGAAAAAAAAAAGTT	This work
DOWN-SIC1-R	CTCCCTTCTTCAAGTTTTAC	This work
UP-ACE2-F	GAATAATGAAAACGATGATGGCCTC	This work
UP-ACE2-R	TCATCTGGTAAAGGAGACGTTGCTTCTCCAATTTTTCGTT	This work
DOWN-ACE2-F	AACGAAAAATTGGAGAAGCAACGTCTCCTTTACCAGATGA	This work
DOWN-ACE2-R	TTCCTCCATTTTCATTAGGACGCTC	This work
CLN3-F	GTTCCTTATTCAGTTAGCTAGCATGGCCATATTGAAGGAT	This work
CLN3-R	GCCTGACTACGCATGATATCTCAGCGAGTTTTCTTGAGGT	This work
SIC1-F	TCCTTATTCAGTTAGCTAGCATGACTCCTTCCACCCCACC	This work
SIC1-R	GCCTGACTACGCATGATATCTCAATGCTCTTGATCCCTAG	This work

ACE2-F	TCCTTATTCAGTTAGCTAGCATGGATAACGTTGTAGATCC	This work
ACE2-R	GCCTGACTACGCATGATATCTCAGAGAGCATCAGTTTCGT	This work

Table S2 Genes and primers used for qRT- PCR

Gene	Forward primer sequence (5′-3′)	Reverse primer sequence (5′-3′)
CLN1	AAAACTTGGGGAGGGTGCAA	GACCCGCCGCAATAATGAAC
CLN2	GATGTATCCGCGTGGCAGTA	GGACCGTGGTCTTGATTGGT
CLB5	GCTGGTAGAGGTGCACGAAA	TTGCCGCGATGAAAAGTGAG
CLB6	GGACACGCAATCTCCCTATCA	AGGCAATGAACAGGCAAGTGA
CLB3	TAGTTGGTTAGCTGCCGGTG	ATGGTGGCCAACGGAAGAAT
CLB1	GCCTTCTGCCGGAAACTCTA	CATGCACCGTCTGTCTCGTA
CLB2	GGTTGGCACATCATGCCTTT	TTCCGTACATGCACCGTCTG
PCL2	ATCGCAAAGAGACTGTCGCA	GAGAGGGTCGAAGTGGAAGC
PCL9	ACACTGGACCACGTACACAG	TCCTTGATAGGCCCGAGGAA
FLO1	AACCGCGTTCAACTGTTGTG	AACCGCGTTCAACTGTTGTG
FLO5	TGGTTACCGTGACATCCTGC	TGTGACGCCGCTAACAGTAA
FLO11	CCGCTGGTAAGACGACAACT	TGGTACGGCATTAGTGGCAG
FKS1	ACCTTATCAGGGCCAAACGG	CAGCAGCGACATTTGGATCG
FKS2	TATGTTGGGGCGAGGCAAAT	CCTTCAGGCAAGGGATCAGG
FKS3	TTATGACCACTCGTGGTGGC	CACGGCAGATTGCGTTCATT
TPS1	TAACGCTAAGGCGCAACTGA	CTCGTACTGTCCCGTACTGC
TPS2	TCCAAAGTGCTGGTACGGTC	CTTTGCGCCGGTGTAAGAAG
TPS3	TACAACAGTGGCGTCAGTCC	GAACCGGAAACGCAAACGAA
CLN3	TTGACCGCGCTTTGGATTTC	TCGAATGTTGCCGACTGACA
SIC1	GCTTACGTCTCCTCAACGCT	CACATTTTGCTGCGTGGGAA
ACE2	CACATTTTGCTGCGTGGGAA	TGGGTTCAGTGTCATCCTGC
